# Occurrence and Clinical Outcomes of Acute Kidney Injury (AKI) Secondary to Acute Pyelonephritis in Hospitalized Patients at Dubai Health Facilities: A Retrospective Study

**DOI:** 10.7759/cureus.101845

**Published:** 2026-01-19

**Authors:** Rehana Hikman Ud Din, Maies S Al-Toubat, Khadija A Hafidh

**Affiliations:** 1 Internal Medicine, Dubai Health, Dubai, ARE

**Keywords:** acute kidney injury, acute pyelonephritis, acute urinary tract infection, outcomes of acute pyelonephritis, rifle criteria

## Abstract

Acute Kidney Injury (AKI) is a rare but significant complication associated with acute pyelonephritis, which, if left untreated, can cause severe renal impairment, thereby affecting renal structure and function.

Upon reviewing the literature, we found that there are limited studies published on the outcomes of acute pyelonephritis. Our study, therefore, aims to evaluate the incidence and clinical outcomes of AKI in patients hospitalized with acute pyelonephritis at tertiary care hospitals under the 'Dubai Health' Institution.

We reviewed the medical health records of patients, noting details such as demographic data, risk factors, and vital signs. Out of 303 patients who were hospitalized with acute pyelonephritis, a marked percentage of patients developed AKI. The incidence of acute pyelonephritis (APN) was higher in the female population than in males; however, the incidence of AKI was higher in males than in females, with a median age of 41 years. A strong association between having a positive blood culture and the development of AKI was noted, thereby denoting the fact that patients with bacteremia were more susceptible to AKI. Chronic kidney disease (CKD), current or recent urinary tract catheterization, and emphysematous pyelonephritis were found to be the commonest risk factors for the development of AKI. Outcomes showed that a significant percentage of patients experienced complete resolution of AKI, a minority progressed to CKD, and perhaps only a small percentage succumbed to the illness.

AKI is a serious but potentially reversible complication of acute pyelonephritis that requires early recognition and prompt management to prevent long-term renal failure. The Risk, Injury, Failure, Loss, and End-stage (RIFLE) criteria serve as a valuable tool in guiding treatment decisions, particularly regarding the need for renal replacement therapy. Older age, male gender, bilateral involvement, and pre-existing CKD were identified as key risk factors, emphasizing the need for close monitoring and timely antibiotic and supportive therapy in these patients.

## Introduction

An acute infection of the urinary tract is one of the commonest case presentations that healthcare providers encounter in a hospital setting. Acute urinary tract infection is an umbrella terminology that covers two clinical presentations, i.e., uncomplicated cystitis that involves an infection of the urinary bladder and complicated urinary tract infections (UTI) that refers to an infection of the upper part of the urinary tract, including the ureters, the parenchyma of the kidney (also called pyelonephritis), or systemic involvement [1]. 

There is no available data on the incidence rates of acute pyelonephritis in the UAE. However, a study conducted in the Middle East and North Africa (MENA) region from 1990 to 2021, reported in February 2025, shows an incidence rate of 4,033.4 cases of UTIs per 100,000 people in 2019 [2]. Acute complicated UTI is more common in the female population than in the male population, most likely due to anatomical differences. Women are more prone to this condition because of the short urethra, which makes it easier for bacteria to ascend to the urinary bladder and subsequently to the kidneys.

Patients with acute complicated UTI present with fever (>37.7°C), chills or rigors, fatigue, flank pain, pelvic or perineal pain, and/or symptoms of acute uncomplicated cystitis, such as dysuria, urinary frequency, urinary urgency, and suprapubic pain. Upon physical examination, costovertebral angle tenderness is elicited. An acute complicated UTI may also present with bacteremia, causing septicemia or septic shock with multi-organ dysfunction, including acute kidney injury (AKI) [1]. This is more likely to happen in the older age group population, female gender, individuals with risk factors like diabetes mellitus, hypertension, dyslipidemia, prior history of UTI, congenital renal disease, urinary tract obstruction, prior urinary catheterization, immunocompromised patients, pregnant women, or known to have chronic kidney disease (CKD) [3].

AKI has been noted to be one of the important clinical outcomes in patients with acute complicated UTI. It is a poor prognostic factor in patients with sepsis [4]. According to the Kidney Disease Improving Global Outcomes (KDIGO) guidelines, AKI is defined by an increase in serum creatinine by ≥0.3 mg/dL within 48 hours, or an increase to ≥1.5 times the baseline level within the prior seven days, or a urine output of <0.5 mL/kg/h for six hours [5]. The concept of the Risk, Injury, Failure, Loss, and End‑stage renal disease (RIFLE) classification system, introduced in the early 2020s, categorizes AKI into the five broad stages [6].

In our study, we outline the occurrence, risk factors, and clinical outcomes of AKI secondary to acute pyelonephritis using the ‘RIFLE’ classification system, as timely diagnosis and management of acute pyelonephritis have a significant impact on patient outcomes and long-term prognosis.

## Materials and methods

We conducted a retrospective study that included 303 patients who were hospitalized with acute pyelonephritis at three tertiary care hospitals within ‘Dubai Health’ between January 2022 and December 2024. Eligible participants were adults aged 18 to 65 years. Patients who were pregnant or immunocompromised were excluded from the analysis. 

Data collected included patient demographics, comorbidities, history of urinary tract infections, urinary tract obstruction, urinary catheterization or instrumentation, urine cultures, and blood cultures. Patients who presented with septic shock were also identified. These were extracted from the hospitals’ electronic medical records and securely stored in an encrypted Excel Spreadsheet (Microsoft Corporation, Redmond, Washington, USA). The study protocol was approved by the Institutional Review Board (IRB) and the hospital ethics committees. Since this is an observational study, the institutional ethics committee waived patients' consent.

AKI was defined according to established criteria as outlined above. CKD was described as a glomerular filtration rate (GFR) of <60 mL/min/1.73 m² [6]. To assess the incidence and clinical outcomes of AKI, patients were stratified into two groups: those who developed AKI and those who did not. Subsequently, patients were further categorized (based on renal recovery) into those who developed CKD and those who regained baseline kidney function, to assess the relationship between AKI severity (as defined by the RIFLE classification) and clinical outcomes.

The RIFLE criteria were applied to classify AKI severity. According to RIFLE criteria, the “Risk” category is defined by a rise in serum creatinine to 1.5 times baseline, a GFR decline of 25%, or a decrease in urinary output to <0.5 mL/kg/hr for six hours. The “Injury” category is defined by a rise in serum creatinine two times from the baseline, a GFR decline of 50%, or a decrease in the urinary output of <0.5mL/kg/hr for 12 hours, “Failure” is defined by a rise in serum creatinine three times from the baseline, a GFR decline of 75%, or a decrease in urinary output of <0.5 mL/kg/hr for 12 hours, “Loss” is defined by a loss of renal function for more than a month and "end-stage renal disease (ESRD)” as a loss of renal function for more than three months [5]. Baseline serum creatinine values were used for classification. The most recent serum creatinine level and eGFR recorded before hospitalization were considered the baseline value. If baseline values were unavailable, the upper limits of the normal reference ranges for serum creatinine and eGFR were used as surrogates. Urine output criteria were not utilized, as accurate measurements were not consistently available upon admission.

Continuous variables were expressed as mean ± standard deviation (SD), while categorical variables were presented as percentages. The Pearson Chi-square test was used to analyze associations between categorical variables. All statistical analyses were performed using IBM SPSS Statistics, version 28 (IBM Corp., Armonk, New York, USA). A p-value of <0.05 was considered indicative of statistical significance. The odds ratio test was also used to assess the association between risk factors for AKI and its clinical outcomes. 

## Results

Among 303 patients admitted with acute pyelonephritis, 125 (41.3%) developed AKI. Based on the RIFLE classification system, 65 (21.5%) patients fall in the “Risk” category, 30 (9.9%) in the “Injury” category, 24 (7.9%) in the “Failure” category, 2 (0.7%) in the “Loss” category, and 5 (1.7%) in the “ESRD” category.

The incidence of AKI was significantly higher in males compared to females; p=0.019, with the median age among patients with AKI being 41 years; p=0.007. The median hospital stay in these patients was six days. Upon presentation to the hospital, 19 (59.4%) patients were in a state of septic shock. Unilateral pyelonephritis was observed in 57 (32.8%) patients with AKI, whereas 68 (52.7%) had bilateral pyelonephritis; p=0.001.

Among all patients with pyelonephritis, i.e., a total of 303 patients, 136 (44.9%) had a positive urine culture. Among those with a positive urine culture, 51 (16.8%) had E. *coli,* 52 (17.2%) had ESBL E. *coli*, 14 (4.6%) had Klebsiella, seven (2.3%) had ESBL Klebsiella, four (1.3%) had Pseudomonas, and nine (3%) had Candida species. Consequently, 59 (43.4%) patients with positive urine culture were noted to have developed an AKI (Table 1). 17(33.3%) patients with E. *coli*, 26 (50%) with ESBL E. c*oli*, seven (50%) with Klebsiella, three (42.9%) with ESBL Klebsiella, and three (33.3%) with Candida positive urine cultures developed AKI. By contrast, all patients who grew Pseudomonas on their urine cultures developed AKI. Overall, there is no association noted between having a positive urine culture and the development of AKI.

**Table 1:** Cross tab showing 59 (43.4%) patients with positive urine culture developed an acute kidney injury (AKI)

			AKI		
			0	1	Total
Urine Culture	0	Count (% within urine culture)	101 (60.5%)	66 (39.5%)	167 (100.0%)
	1	Count (% within urine culture)	77 (56.6%)	59 (43.4%)	136 (100.0%)
Total		Count (% within urine culture)	178 (58.7%)	125 (41.3%)	303 (100.0%)

Among all patients with pyelonephritis, 76 (25.1%) had a positive blood culture. Among those with a positive blood culture, 28 (9.2%) had E. *coli*, 31 (10.2%) had ESBL E. *coli*, 10 (3.3%) had Klebsiella, one (0.3%) had ESBL Klebsiella, two (0.7%) had Pseudomonas, and one (0.3%) had Candida Species. Consequently, 45 (59.2%) patients with a positive blood culture were noted to have developed AKI Table 2. Fourteen (50%) patients with E. *coli* and seven (70%) with Klebsiella developed AKI. On the contrary, all patients who grew Pseudomonas and Candida species on their blood cultures developed AKI. 20 (64.5%) patients who grew ESBL E. *coli* developed AKI (p=0.005); in contrast, no patients with ESBL Klebsiella developed AKI. Overall, there is a strong association between having a positive blood culture and the development of AKI, with a significant p-value of <0.001.

**Table 2:** Cross tab showing 45 (59.2%) patients with positive blood culture developed an acute kidney injury (AKI)

			AKI		
			0	1	Total
Blood Culture	0	Count (% within blood culture)	146 (64.6%)	80 (35.4%)	226 (100.0%)
	1	Count (% within blood culture)	31 (40.8%)	45 (59.2%)	76 (100.0%)
Total		Count (% within blood culture)	177 (58.6%)	125 (41.4%)	302 (100.0%)

AKI developed in 87% of patients with pre-existing CKD; p<0.002. Among those with AKI, 65 (53.7%) were diabetic, 27 (57.4%) were hypertensive, and 22 (59.5%) had dyslipidemia. A prior history of urinary tract infection (UTI) was present in 36 (49.3%) patients, while seven (58.3%) had congenital renal disease. Ureteric obstruction was identified in 27 (54%) cases; p=0.044. All patients (100%) who were catheterized developed AKI, and 11 (37.9%) had a prior history of urological instrumentation. On presentation, 19 (59.4%) of those who presented with initial signs of shock had developed AKI throughout their hospital stay; p=0.029. 44.8% and 50% had renal abscesses and retroperitoneal abscesses, respectively. 84.6% had emphysematous pyelonephritis, 25% had thrombosis, and 50% had DKA. In addition, one (100%) patient who had developed papillary necrosis eventually developed AKI, and only one (0.3%) patient who developed AKI underwent nephrectomy.

Of the patients who developed AKI, in 101 (80.8%) patients it resolved, 20 (16.0%) developed CKD, and three (2.4%) died; p=0.001 Table 3. Dialysis requirements during hospital stay varied across RIFLE categories: 1.5% in the "Risk" group, 10% in the "Injury" group, 4.2% in the "Failure" group, 0% in the "Loss" group, and 40% in the ESRD group; p<0.001 Table 4.

**Table 3:** Cross-tabulation table showing outcomes of acute kidney injury (AKI)

			Outcomes				
			0	1	2	3	Total
AKI	0	Count (% within AKI)	31 (17.4%)	1 (0.6%)	146 (82.0%)	0 (0.0%)	178 (100.0%)
	1	Count (% within AKI)	101 (80.8%)	20 (16.0%)	1 (0.8%)	3 (2.4%)	125 (100.0%)
Total		Count (% within AKI)	132 (43.6%)	21 (6.9%)	147 (48.5%)	3 (1.0%)	303 (100.0%)

**Table 4:** Cross-tabulation table illustrating dialysis requirements during hospital stay varied across Risk, Injury, Failure, Loss, and End-stage (RIFLE) categories

			Underwent Dialysis		
			0	1	Total
Severity of AKI (RIFLEs classification)	0	Count (% within severity of AKI [RIFLEs classification])	176 (99.4%)	1 (0.6%)	177 (100.0%)
						1	Count (% within severity of AKI [RIFLEs classification])	64 (98.5%)	1 (1.5%)	65 (100.0%)
	2	Count (% within severity of AKI [RIFLEs classification])	27 (90.0%)	3 (10.0%)	30 (100.0%)					
						3	Count (% within severity of AKI [RIFLEs classification])	23 (95.8%)	1 (4.2%)	24 (100.0%)
	4	Count (% within severity of AKI [RIFLEs classification])	2 (100.0%)	0 (0.0%)	2 (100.0%)					
						5	Count (% within severity of AKI [RIFLEs classification])	3 (60.0%)	2 (40.0%)	5 (100.0%)
	Total		Count (% within severity of AKI [RIFLEs classification])	295 (97.4%)	8 (2.6%)						303 (100.0%)

## Discussion

We reviewed 303 patients retrospectively, admitted from January 2022 to December 2024, with acute pyelonephritis (APN) at tertiary care hospitals under ‘Dubai Health’.

The overall incidence of AKI in patients admitted with APN was 41.3%, with the median age being 41 years. In a similar study that was conducted in Northern India, the incidence of AKI in patients with APN was 83.2% [7]. Recent cross-sectional studies conducted in Karachi and the West Indies reported an incidence rate of 58.2% and 64%, respectively [3,8]. The lower incidence rate of AKI in our study may be due to early hospital presentation and early detection of the condition. The incidence of APN was significantly higher in the female population as compared to the males, owing to the cause being the anatomy of the female urinary system, making them more prone to UTIs; however, the risk of AKI was seen to be higher in the male population, with a significant p-value of 0.019. Similar results of increased incidence rates of AKI were reported in multiple studies conducted in Asian countries [4,7]. The duration of hospital stay was also longer for patients with AKI.

We applied the RIFLE classification criteria to further categorize patients with AKI, which not only helps to determine the modality of treatment but also to trace outcomes. Analysis showed that 21.5% fell into the “Risk” category, 9.9% into the “Injury” category, 7.9% into the “Failure” category, 0.7% into the “Loss” category, and 1.7% into the “ESRD” category. Another study that was conducted recently in the West Indies in 2023 also showed a higher percentage of patients falling in the Stage 1 category (mild AKI) according to the KDIGO classification system [8]. However, a similar study that was conducted in South Korea in 2019 showed a higher percentage of patients in each category, i.e., “Risk”, “Injury”, and “Failure”, with results from our study being comparatively better [4]. 

Univariant analysis showed that diabetes mellitus, hypertension, dyslipidemia, history of urinary tract obstruction, urinary catheterization, initial presentation of shock, positive blood culture and emphysematous pyelonephritis were significant risk factors for AKI, whereas, multi-variant analysis showed that older age, male gender, patients known to have CKD and patients having bilateral pyelonephritis were independent risk factors associated with AKI, therefore bringing us to a conclusion that multiple overlapping risk factors influence the development of AKI. Similar findings were reported in the study conducted in the West Indies, showing an increased incidence of AKI in patients >65 years of age, patients known to have hypertension, and those with underlying CKD [8]. On the other hand, a retrospective cohort study that was conducted in Korea that studied the effects of urolithiasis-related obstructive uropathy duration and concomitant APN and AKI on the renal outcomes, showed that early release of obstruction led to an improvement in renal outcomes [9].

Patients who were known to have CKD had a higher frequency (87%) with a significant p-value of <0.002 and severity of AKI; however, our study showed no increase in the rate of mortality in such patients and instead showed a return to baseline renal function upon initiation of appropriate treatment. Similar findings were reported in studies from South Korea, North India, and Denmark [4,7,10].

Urine cultures were positive in 136 (44.9%) patients with APN, with E. *coli* and ESBL E. *coli* being the most common organisms isolated. Similar findings were reported in the study conducted in the West Indies [8]. Consequently, 59 (43.4%) patients with positive urine cultures, 17 (33.3%) with E. *coli*, and 26 (50%) with ESBL E. *coli* developed AKI; however, this did not reach statistical significance. Similar results were noted in the study from North India [7]. On the other hand, blood cultures were positive in 76 (25.1%) patients with APN, with E. *coli* and ESBL E. *coli* being the most common organisms isolated. Consequently, 45 (59.2%) patients with positive blood cultures developed AKI (p-value of <0.001), indicating strong statistical significance.

With supportive care and initiation of appropriate antibiotics, 101 (80.8%) patients had resolution of AKI. 20 (16.0%) patients with AKI went into developing CKD. Three (2.4%) patients died, with cardiogenic shock being the cause of death in one patient, septic shock being the cause of death in two patients, and, more commonly in the older age group and male population, severe AKI, and with bilateral pyelonephritis. On the other hand, the requirement for hemodialysis varied across RIFLE categories, with patients in the “Injury” category (10%) and "End-Stage Renal Disease (ESRD)" category (40%) being the highest group of patients requiring hemodialysis.

Declining renal function secondary to AKI significantly increases morbidity and mortality. Some of the complications associated with severe AKI include volume overload, especially in patients with heart failure; lung impairment; hyperkalemia, and disturbance of acid-base homeostasis [11]. In a cohort study that was performed on Medicare patients, AKI was associated with a 40-fold increase in the development of ESRD in patients with AKI on top of CKD, as compared to AKI alone and the same was reported in our study thereby facilitating the need for early recognition and management of AKI especially in high risk group patients like those with underlying CKD [11]. According to a prospective study that was conducted at a tertiary care hospital in North India, the most common sepsis-related etiology of AKI included urosepsis; therefore, timely administration of antibiotics significantly improves outcomes [12]. On the other hand, in another study that was conducted in Chennai, India, it was reported that DJ stenting in addition to conservative management and antibiotics in patients with non-obstructive APN brought about a significant improvement in renal outcomes, especially in cases of Stage 3 AKI [13]. 

Although our study highlighted important factors in the development of AKI in patients with APN, there are some limitations to take into consideration. Firstly, our research was limited to the population of Dubai, therefore the results do not represent the incidence of AKI in patients with APN in the U.A.E. Secondly, flank pain is an important symptom in patients with APN, which not only is an important diagnostic but also a prognostic factor, however, it was not recorded due to multiple factors like communication barrier, underlying dementia, hypoesthesia and unstable patient presentation to the hospital, which was a limitation of our study [4]. Moreover, this is a retrospective study, and information was extracted from the medical health records of patients; therefore, some information, like administration of nephrotoxic agents or use of contrast media, which is again a vital add-on risk factor to the development of AKI in patients with APN, before hospital presentation, might have been missed. Furthermore, when considering the RIFLE criteria, we used serum creatinine and eGFR values to determine renal involvement stage, as urine output wasn’t available for all patients. Lastly, there was no follow-up visit for patients or repeat renal function tests after discharge from the hospital to trace the recurrence of AKI. To overcome these limitations, we suggest conducting further research on this topic to provide a broader picture of the disease and its prognosis.

## Conclusions

AKI is a complication of APN that requires prompt attention to avoid the development of renal failure and subsequent dependence on renal replacement therapies in the long run. Though a serious complication of APN, it is yet a reversible condition with early initiation of management, and the RIFLE criteria have been noted as an excellent guide towards patient management, especially in determining patients who need renal replacement therapy in the form of hemodialysis. Old age, male gender, bilateral APN and CKD are significant risk factors towards the development of AKI; therefore, patients with such risk factors need more attention. Overall, initiation of appropriate antibiotic therapy and supportive care, including intravenous fluid replacement therapy, played an essential role in managing both APN and AKI.

## References

[1] (2025). Complicated urinary tract infections (CUTI): clinical guidelines for treatment and management. https://www.idsociety.org/practice-guideline/complicated-urinary-tract-infections/.

[2] Amiri F, Safiri S, Aletaha R, Sullman MJ, Hassanzadeh K, Kolahi AA, Arshi S (2025). Epidemiology of urinary tract infections in the Middle East and North Africa, 1990-2021. Trop Med Health.

[3] Soniya Soniya, Jabeen R, Guriya Guriya, Gul L, Ejaz A, Kashish Kashish (2025). Frequency of acute kidney injury in patients presenting with acute pyelonephritis at tertiary care hospital, Karachi. Indus Journal of Bioscience Research.

[4] Jeon DH, Jang HN, Cho HS, Lee TW, Bae E, Chang SH, Park DJ (2019). Incidence, risk factors, and clinical outcomes of acute kidney injury associated with acute pyelonephritis in patients attending a tertiary care referral center. Ren Fail.

[5] Abhinav Goyal; Parnaz Daneshpajouhnejad; Muhammad F. Hashmi; Khalid Bashir (2023). Acute Kidney Injury. Treasure Island (FL): StatPearls Publishing; 2025 Jan..

[6] Lopes JA, Jorge S (2013). The RIFLE and AKIN classifications for acute kidney injury: a critical and comprehensive review. Clin Kidney J.

[7] Seth S, Bhat NA, Sheikh RY, Keshwani P, Mehta P (2023). Occurrence and risk factors for acute kidney injury in patients hospitalized with acute pyelonephritis, and their clinical outcomes: a single center study from Northern India. APIK Journal of Internal Medicine.

[8] Hoe KK, Reid T (2025). Prevalence, risk factors and outcomes of acute kidney injury among hospitalized patients with urinary tract infection a retrospective cross-sectional study. Int J Nephrol Kidney Fail.

[9] Lee EH, Kim SH, Shin JH, Park SB, Chi BH, Hwang JH (2019). Effects on renal outcome of concomitant acute pyelonephritis, acute kidney injury and obstruction duration in obstructive uropathy by urolithiasis: a retrospective cohort study. BMJ Open.

[10] Graversen HV, Nørgaard M, Nitsch D, Christiansen CF (2021). Preadmission kidney function and risk of acute kidney injury in patients hospitalized with acute pyelonephritis: a Danish population-based cohort study. PLoS One.

[11] Almazmomi MA, Esmat A, Naeem A (2023). Acute kidney injury: definition, management, and promising therapeutic target. Cureus.

[12] Tahir S, Ganie BA, Beigh TY, Hazar AJ, Reshi AR (2023). Clinico-etiological spectrum and outcome in patients with septic acute kidney injury and its comparison with non-septic acute kidney injury: a hospital-based prospective study conducted in a tertiary care hospital in North India. Cureus.

[13] Palliyedath R, Prabahar M R, Jayam J, Sivanandam S, Veeramani M, C N (2025). The role of double j stenting in nonobstructive acute pyelonephritis with acute kidney injury. Cureus.

